# Where do cross-cutting discussions happen?: Identifying cross-cutting comments on YouTube videos of political vloggers and mainstream news outlets

**DOI:** 10.1371/journal.pone.0302030

**Published:** 2024-05-29

**Authors:** Seung Woo Chae, Sung Hyun Lee

**Affiliations:** 1 Department of Information & Library Science in the Luddy School of Informatics, Computing and Engineering, Indiana University, Bloomington, Indiana, United States of America; 2 The Media School, Indiana University, Bloomington, Indiana, United States of America; Universidade Federal de Ouro Preto, BRAZIL

## Abstract

Since the conception of social media, research on political communication has pointed toward the risk that the social media environment can foster political echo chambers. However, this has recently been contradicted by some studies demonstrating “cross-cutting discussions” on social media. The current study extends this literature by particularly focusing on communication on political vlogger videos and having mainstream news outlet videos as a reference point. Specifically, this study addresses five points: (1) to what extent cross-partisan comments occupy conservative and liberal vloggers’ comment threads and if there is a significant difference between the two, (2) the possibility that comments from vlogger videos can be utilized to predict the political leanings of comments on mainstream news outlet videos, (3) if the proportion of cross-cutting discussions on mainstream news outlet videos significantly varies by the news outlet’s political leaning, (4) if a neutral news outlet channel can work as a venue for cross-cutting discussions, and (5) if the proportion of cross-cutting comments in mainstream news outlet comment threads is significantly different from that in vlogger comment threads. Both manual and computational analyses were employed; the political leanings of vlogger comments were analyzed by manual content analysis, and based on the results, the political leanings of mainstream news outlet comments were analyzed by NLP classifiers using three different algorithms—logistic regression, SVM, and random forest. As a result, we found that the proportion of cross-cutting discussions significantly varies by both the channel’s political leaning and media type. In addition, our results suggest the possibility of neutral news outlets as a place for cross-cutting discussions.

## Introduction

Previous studies have found that individuals tend to consume information in line with their existing ideas instead of against them [1,2]. This phenomenon is not necessarily harmful, but rather can seem natural. Prior literature often explains this phenomenon with the concept of “selective exposure,” which refers to individuals’ tendency to attend to information that is aligned with their preconceived views [3], and considers it as one of the common tendencies that individuals can have. However, in the context of political communication, it is a subject of concern that can induce people to distort their perception of reality and make them politically biased. Some concepts, including “echo chamber,” which represents an environment in which a person’s beliefs, ideas, or opinions are reinforced by being exposed only to information that aligns with their existing views, were developed to caution people about their biased news consumption [4,5].

These concerns about political echo chambers increased especially after the emergence of the Internet [6]. No doubt, the online environment has drastically changed the way that people consume their news. Now, access to a multitude of online news articles is available mostly without time and location restrictions, and most of the articles are *free*, which was unimaginable back when we consumed news from physical sources. The prevalence of social media has changed our patterns of news consumption even more. Above all, social media has diversified the everyday news sources from which we gain information. It is currently a common practice, especially among young people, to learn new facts from their friends via social media. In other words, today, the people we are connected to on social media can determine the kind of information we receive. As such, researchers have argued that the current trend of online news consumption via social media can reinforce echo chamber effects [7,8].

However, this argument is not conclusive. There have been an increasing number of studies that empirically refute it and suggest that the digital environment does not instigate echo chambers [9,10]. A concept often used in their refutation is “cross-cutting discussion” [11], which describes a form of dialogue or conversation that involves individuals from diverse backgrounds, with differing perspectives, opinions, or affiliations, engaging in open and constructive communication about a particular topic or issue [12]. Cross-cutting discussions on social media are often employed in literature as proof to contradict the argument of online echo chamber effects [13]. This rebuttal has become more compelling especially as some researchers have conducted large-scale data analysis via state-of-the-art computational methods [e.g., 14]. Presently, the two threads of research that support [7,8] and refute [10,11] the notion of online echo chambers have continued, and the jury is still out.

The current study shares our perspective on this debate and extends the literature by complementing it with a specific focus on communication on political vloggers’ videos; throughout this paper, we define political vloggers as people who regularly upload videos related to politics on social media based on their political beliefs. In addition, we also explore mainstream news outlet videos as a reference point for these vlogger videos. Specifically, this study addresses five points by analyzing comments from the vlogger and mainstream news videos on YouTube: (1) to what extent cross-partisan comments occupy conservative and liberal vloggers’ comment threads and if there is a significant difference between the two, (2) the possibility that comments from vlogger videos can be utilized to predict the political leanings of comments on mainstream news outlet videos, (3) if the proportion of cross-cutting discussions on mainstream news outlet videos significantly varies by the news outlet’s political leaning, (4) if a neutral news outlet channel can work as a place for cross-cutting discussions, and (5) if the proportion of cross-cutting comments in mainstream news outlet comment threads is significantly different from that in vlogger comment threads. We conducted both manual and computational analyses; the political leanings of vlogger comments were analyzed by manual content analysis, and based on the results, the political leanings of mainstream news outlet comments were analyzed by natural language processing (hereinafter NLP) classifiers.

In what follows, we will first discuss relevant literature and propose the research questions we drew from it. Next, the materials and methods section will introduce our two different samples, which are comments drawn from vlogger and mainstream news videos on YouTube, respectively. In that same section, we will then delineate how we analyzed the political views of those comments. After that, we will exhibit the results related to each research question. The meanings of these results will be discussed in the context of political communication and NLP literature in the discussion section. Lastly, our conclusion will summarize the overall study and reiterate the main points of our findings.

## Literature review

### Echo chamber and cross-cutting discussion

The early use of “echo chamber” as a metaphorical term dates back to the 20^th^ century [15]. While it has been used in various contexts since then, it is after the beginning of the current century that researchers started to increasingly use the term to specifically indicate exclusive exposure to opinion-reinforcing information. In his book *Republic*.*com* [6] published in 2001, Sunstein introduced the idea of individuals being confined to informational echo chambers where their views are reinforced back to them. Following that, with the concept of echo chamber, Jamieson and Cappella [5] argued that increased channels for news content strengthen people’s behavior to seek news outlets consistent with their political leanings. They mentioned Rush Limbaugh’s radio talk show, Fox News, and *The Wall Street Journal* as examples that formed an echo chamber maintaining political conservatism and reinforcing the phenomenon of selective exposure. This thread of research has been well extended until now, and currently, the echo chamber effect is one of the most actively explored topics in political communication [16,17].

Contemporary literature on the echo chamber effect particularly points to digital algorithms as its core cause [7]. Many recommendation algorithms on social media platforms are designed to recommend similar content to what was previously seen, and their feed algorithms explicitly foster an environment where people stick with their existing news sources [18,19]. Consequently, some studies have argued that these algorithms can induce people to encounter information mostly aligned with their preexisting ideas and that, within their online echo chamber, political partisanship can solidify to the point where they believe that their political group’s ideas are the only truth [20]. Particularly in the context of U.S. politics, which the current study focuses on, these concerns have resonated with many people because political polarization has been a serious issue, especially since the 2016 presidential election [21], as demonstrated by the January 6 U.S. Capitol Attack.

That said, the argument that the digital environment instigates echo chambers is not conclusive. Garrett ignited the debate with his empirical study [9] examining the existence of online echo chambers. He recruited reader groups of two partisan online news sites, which represented liberalism and conservatism, respectively, and tracked their news consumption behaviors. The results revealed that when people consume online news, there is no evidence that they avoid news content that they disagree with. While the participants in the web-administered study were more likely to look at opinion-reinforcing news rather than opinion-challenging news, regarding the time they spent, they spent more time looking at the opinion-challenging news than the other. As mentioned earlier, lately, the concept of “cross-cutting discussion” has been frequently employed to support the argument of this side [13]. However, it would be more precise to say that cross-cutting discussions can not only question the seriousness of online echo chambers but also be a solution to those echo chambers, which can mitigate political bias by exposing people to opinion-challenging ideas.

Of the studies about cross-cutting discussions, Wu and Resnick’s recent study [14] is particularly compelling. Using computational methods, the authors selected over 274,000 YouTube videos from 973 U.S. partisan media channels and collected 134 million comments from more than 9 million users. With this large-scale dataset, they tested the extent of cross-cutting discussions on liberal and conservative sides, respectively. The results displayed asymmetric cross-cutting discussions across political groups; 29.5% of comments on left-leaning videos were written by conservatives, while 13.4% of comments on right-leaning videos were written by liberals. This empirical finding of cross-cutting discussions from big data begs the question of whether echo chambers on social media are a serious issue. Additionally, the study investigates how the proportion of cross-cutting discussions varies by *media type*. They found that cross-cutting discussions are more frequently shown on mainstream news outlet channels (e.g., CNN, Fox News), whereas independent news media channels showed relatively low cross-cutting rates. This new approach suggested the possibility that online echo chamber effects can vary by people’s preferred media type.

### The current study

The current study extends this cross-cutting discussion literature by complementing it with a specific focus on communication on political vloggers’ videos. While Wu and Resnick’s approach to media type was novel [14], their categorization was not ideal for seeing how communication on political vlogger videos is different from communication on other media types; their category of “independent media” included both organizations (e.g., The Young Turks, Breitbart) and individual vloggers (e.g., Ben Shapiro, Paul Joseph Watson). Accordingly, the study did not exhibit concrete results specifically about political vloggers. We recognize this gap in the literature as a spot where the current study can contribute, considering the enormous influence of political vloggers in the current landscape of political communication. In terms of their influence on social media users, popular political vloggers are comparable to mainstream news outlets. For instance, the political vlogger Steven Crowder has 5.84 million subscribers on YouTube as of July 2023, which is higher than the number of subscribers to CNBC News (5.08 million). Furthermore, some political vloggers have even launched their own news outlets based on their popularity and have attracted many viewers, as we can see in the case of Tim Pool’s *Timcast*.

Not only the quantity, but also the quality of communication on political vloggers’ channels is worth academic attention. Recent work in social media research has pointed toward parasocial relationships between social media influencers and their followers [22–24], in which users perceive influencers to be akin to their friends in the real world [25]. Particularly on YouTube, lately, there have been multiple studies that explain the relationship between vloggers and their regular viewers via this concept. Lee and Watkins’s work [26] was one of the early studies that explored parasocial relationships between vloggers and their audiences on YouTube. The study tested if vloggers influenced their viewers’ perceptions of luxury brands and found that viewers’ perceptions of luxury brands significantly increased after watching relevant vlogger videos. The authors argued that this association originated from the parasocial relationship between vloggers and their viewers. As a following study, Ferchaud et al.’s study [27] more broadly explored parasocial relationships on YouTube by examining the parasocial attributes of the most subscribed channels on YouTube. This study offered an important framework to measure parasocial relationships on YouTube, which many later studies could expand upon.

Despite this relevant literature, to our knowledge, the question of whether parasocial relationships can develop specifically between political vloggers and their audiences has not yet been addressed. This is somewhat surprising given the fact that now political vloggers are compelling players in political communication and that parasocial relationships are an emerging topic in social media research. As prior literature demonstrates, YouTube viewers in parasocial relationships are likely to follow their persona’s suggestions or ideas [26]. Within the context of political vloggers, we assume that viewers in parasocial relationships tend to become like-minded with their persona, which could eventually hinder cross-cutting discussions. Hence, by probing into the extent of cross-partisan discussions in the comment threads of conservative and liberal vloggers’ videos, the present study aims to extend not only the literature on political communication but also help find clues about parasocial relationships between political vloggers and their audiences. Therefore, we propound the following research question:

*RQ1-1*. To what extent do cross-partisan discussions occupy conservative and liberal vloggers’ comment threads?

Furthermore, we considered it noteworthy that Wu and Resnick [14] found that conservatives are more likely to participate in cross-cutting discussions than liberals on social media. In fact, their study is not the only one that shows asymmetric participation in cross-cutting discussions between conservatives and liberals. Heatherly et al. [13] conducted an online survey to investigate the relationship between social media use and involvement in cross-cutting or like-minded discussions. As a result, they found that conservatives participate in cross-cutting discussions on social media more frequently than liberals. Based on these studies, we test if the same asymmetric pattern exists particularly on political vloggers’ videos. Therefore, we propose another research question as follows:

*RQ1-2*. Does the extent of cross-partisan discussions in a comment thread significantly vary by the vlogger’s political leaning?

Additionally, this study also investigates the methodological utility of comments from political vloggers’ channels. Nowadays, NLP via machine learning is an emerging method for political communication research, and a compelling area in NLP research is text classification, which enables us to distinguish the political leaning of text [e.g., 28–30]. In the process of classifier development, necessary and important resources are training data for machine learning; the quality of training data can heavily influence the overall accuracy of analysis. Accordingly, finding a good source that can provide training data representing the group’s characteristics effectively is essential in supervised text classification. In this sense, if comments on political vlogger videos are relatively more homogeneous in terms of their political leaning, which is a considerable possibility based on prior literature [26], it can be a good source of training data that well represents the typical characteristics of each political group and, in turn, enhances the accuracy of classification. For this purpose, we examine how well comments from political vlogger videos can be used to predict the political leanings of comments from another source, which is mainstream media in this study.

*RQ2*. How well can comments from vlogger videos work as training data to predict the political leanings of comments from mainstream news outlet videos?

With an analysis of comments on mainstream news outlet videos, we also ascertain if the frequency of cross-cutting discussions is asymmetric between conservative and liberal mainstream news outlet videos. Again, Heatherly et al. [13] found there to be asymmetric interests in cross-cutting discussions between the two political groups via their survey, and Wu and Resnick’s study [14] empirically confirmed this difference in their YouTube study analyzing large-scale data via NLP. In the current study, we re-examine this asymmetry using a method different from that of the two studies. Moreover, being different from Wu and Resnick’s YouTube study, the current YouTube study includes comments from the channel of a politically neutral news outlet, C-SPAN. Chae and Hara’s recent study [31] affords the possibility that non-partisan channels can work as a venue where conservatives and liberals can naturally meet on YouTube. Building on this finding, we will test the possibility of neutral news outlets to be a venue for cross-partisan discussions by measuring the ratio of conservative and liberal comments in the C-SPAN comment thread. Taken together, the following two research questions are suggested:

*RQ3-1*. Does the proportion of cross-cutting discussions significantly vary between conservative and liberal mainstream news outlet videos?
*RQ3-2*. In what ratio do conservative and liberal comments make up the comment thread of the neutral mainstream news outlet video?

Finally, the current study extends the research on how *media type* is associated with echo chamber effects on social media. By comparing the proportions of cross-cutting discussions between the vlogger and mainstream news outlet videos, we not only exhibit the difference between these two media types but also ascertain Wu and Resnick’s finding that mainstream news outlets have a higher proportion of cross-cutting comments than other types of media [14]. Therefore, we suggest the following as our last research question:

*RQ4*. Is the proportion of cross-cutting comments in mainstream news outlet comment threads significantly higher than that in vlogger comment threads?

## Materials and methods

The analysis of the current study is twofold. First, comments on political vlogger videos were manually analyzed. Second, based on the results of the manual analysis, comments on mainstream news outlet videos were analyzed by text classification models via supervised machine learning.

### Data

All videos and comments used in this study were about a single political issue, the report on the investigation into Russian interference in the 2016 U.S. Presidential Election, which is more commonly called the *Mueller report*. The reason for selecting only one news topic is that it is challenging to identify the political leaning of general text with NLP, as Pang and Lee’s prominent work [32] on opinion mining indicates. The authors argued that part of the difficulties in NLP opinion mining stems from researchers’ tendency to analyze “general attitudes expressed through texts that are not necessarily targeted at a particular issue or narrow subject” [32, p. 19]. Barberá et al. [33] also supported this idea; they investigated the degree of ideological segregation in social media usage and found that, in contrast to our common belief about the strong effects of political ideology, ideological preference is not a decisive index to define people’s opinions across multiple topics. In practice, even state-of-the-art NLP models struggle to classify the political leanings of general text, and it is rather commonsensical that detecting a political group’s homogeneous linguistic pattern in their texts about mixed topics is much more difficult than finding one in their texts about a particular issue.

#### The Mueller report

Based on this consideration, a single political issue that could draw significant linguistic differences between conservatives and liberals was selected as one identical topic across our training and target data. Among the candidate topics that made enough social impacts to be actively discussed on both vlogger and mainstream news outlet channels, the Mueller report was finally selected. There were two main reasons behind this selection. On the one hand, the topic was sensitive enough to divide stances between liberals and conservatives clearly. It was a hot-button issue in early 2019 as it investigated the suspicion that President Trump and Russia had coordinated to intervene in the 2016 U.S. presidential election, and the conclusion of the report could have been a critical threat to the Trump administration. On the other hand, it was not an ongoing issue at the time when the data were collected, which was positive for our analysis. If an ongoing news topic had been selected, we would not have been confident that the discussions were completed and appropriate for analysis.

As a news topic, the Mueller report generated a long series of news reports over years across a wide range of U.S. news media including both mainstream news outlets and political vlogger channels. For our data, the comments under YouTube videos that were uploaded only between Mueller’s report submission to the U.S. Department of Justice on March 22, 2019, and the U.S. Attorney General William Barr’s official statement on the Mueller Report release on April 18, 2019, were collected. After that period, the focus of news coverage about the Mueller report was significantly changed, which could decrease the quality of our analysis. Lastly, it is worth noting that the newly released information about the Mueller report at the time was mostly in favor of President Trump and conservatives as it turned out that the report was not as threatening as liberals had expected.

#### Comments on political vlogger videos

Judgment sampling was applied for selecting sample videos. Initially, the top 50 political vloggers who have the most subscribers were chosen, referring to two websites that track social media statistics, *Feedspot* and *Socialblade*, which prior literature on YouTube employed [34]. Next, the vloggers who did not upload a video about the Mueller report within one week after Mueller’s submission date (March 22, 2019) were excluded. Among the remaining vloggers’ videos related to Mueller’s submission, videos with fewer than 100,000 views or 100 comments were cut out to keep a certain degree of external validity. Lastly, the vlogger’s political leaning was applied as the final filter in order to match the numbers of conservative and liberal vloggers’ videos. As a result, the five most-viewed videos were selected from each of the conservative and liberal video groups, thus the total number of videos became 10 vlogger videos (see Table 1). Their political orientation was very clear, to the point where they had urged their viewers to behave in ways that would support their political side. Correspondingly, these vloggers’ channels tended to appeal particularly to viewers who have the same political leaning, which we found through our qualitative observations of the comments for our sampling. In addition, it is worth mentioning that in our initial stage of the search, we sought to find videos from three different vlogger groups with three different political leanings: conservative, liberal, and neutral. However, we realized that it is practically difficult to find vloggers representing the neutral political leaning as their clear political leaning itself might be the point that can attract viewers.

**Table 1:** Description of vlogger videos.

YouTube Channel	Number of views	Number of comments	Uploaded date	Vlogger’s political leaning
StevenCrowder	982,817	4,970	Mar 25, 2019	Conservative
Mark Dice	626,013	7,739	Mar 23, 2019	Conservative
Paul Joseph Watson	617,447	6,140	Mar 25, 2019	Conservative
The Jimmy Dore Show	218,270	2,969	Mar 23, 2019	Liberal
Hunter Avallone	143,933	1,590	Mar 27, 2019	Conservative
Secular Talk	122,476	2,114	Mar 25, 2019	Liberal
David Pakman Show	67,498	930	Mar 25, 2019	Liberal
The Majority Report w/ Sam Seder	58,769	254	Mar 25, 2019	Liberal
RonPaulLibertyReport	11,000	170	Mar 25, 2019	Conservative
TBTV	3,851	139	Mar 23, 2019	Liberal

*Note*. The number of views and comments were recorded as of July 25, 2023, and the number of comments counted replies.

As Table 1 shows, the videos in the sample were uploaded on YouTube from March 23, 2019, to March 27, 2019. The video with the highest number of comments had 7,739 comments (*Mark Dice*), and the video with the lowest number of comments had 139 comments (*TBTV*), as of July 25, 2023. All of the comments were collected via the YouTube application programming interface (API). To avoid variance over time, data collection was conducted within three hours, using the programming language Python. As previous studies on the Mueller report did [35], replies to other viewers’ comments were excluded from our sample. This exclusion was necessary because even state-of-the-art NLP models could not properly analyze the replies as they have different contexts from the top-level comments (original comments responding to the video), depending on the content of the previous comment that each reply responds to. Finally, for each video, 100 randomly chosen comments were included in the final sample of vlogger comments. That is to say, the final sample size of the training data was 1,000 comments, which were composed of 500 comments from conservative vlogger videos and another 500 comments from liberal vlogger videos.

#### Comments on mainstream news outlet videos

As RQ3-1 tests, this study compares the political leanings of comments on conservative and liberal mainstream news outlet videos. We selected mainstream news outlet videos that specifically focused on the same event: the attorney general William Barr’s press conference on April 18, 2019 about the Mueller report release. None of the videos included any commentary; all of the videos in our sample contained direct footage from the press conference and were all uploaded on the same day (Aug 18, 2019), while their video lengths varied. With this sampling approach, our comparison between comments from different news outlets with distinct political leanings became fairer and more accurate.

Table 2 shows the list of mainstream news outlet videos from which we collected comments. Those videos were from three groups of channels with different political leanings—conservative, liberal, and neutral. Conservative channels included Fox News and LiveNOW from FOX, both of which are owned by the Fox Corporation. Liberal channels included MSNBC and CNN. Lastly, the neutral group included only one channel, C-SPAN. We initially tried to find more than one channel for the neutral group but failed to find more mainstream news outlets that maintain neutrality like C-SPAN. C-SPAN (the Cable-Satellite Public Affairs Network) is a public service channel that mainly broadcasts “gavel-to-gavel proceedings of the U.S. House of Representatives and the U.S. Senate, and to other forums where public policy is discussed” [36, 1^st^ para], mostly without editing or commentary. As Table 2 displays, the Fox News video had both the highest number of views (510,372 views) and comments (2,865 comments), whereas the C-SPAN video had the lowest for both (20,723 views and 354 comments). As with the vlogger comments, all mainstream news outlet comments in our sample were extracted via the YouTube API. Again, replies to other viewers’ comments were excluded, based on prior literature denoting that the inclusion of replies can decrease the accuracy of classification [35]. Finally, a total of 4,230 comments were collected from the seven YouTube videos as our final sample for mainstream news outlet videos.

**Table 2:** Description of mainstream news outlet videos.

Title	News outlet	Number of views	Number of comments
AG Barr holds news conference on release of Mueller report	Fox News	510,372	2,865
WATCH: A.G. William Barr SLAMS Reporter After She Questions Mueller Report	LiveNOW from FOX	401,537	595
Bill Barr pressed about findings in Mueller report	CNN	119,210	1,337
FULL: Mueller Report Released Attorney General William Barr News Conference	LiveNOW from FOX	98,571	930
Barr: Mueller found no Trump campaign collusion	CNN	69,548	1,708
William Barr: Donald Trump Potential Obstruction Probed, Disagreed On Mueller Legal Theory	MSNBC	31,914	338
Attorney General William Barr Statement on Mueller Report Release (C-SPAN)	C-SPAN	20,723	354

*Note*. The number of views and comments were recorded as of July 25, 2023, and the number of comments counted replies.

### Analysis

In both the manual and computational analyses, we calculated the proportion of cross-cutting comments for each political side. Following Wu and Resnick’s operationalization [14], cross-cutting comments were defined in our analysis as comments that showed the opposing political leaning to the YouTube channel’s political leaning. In other words, cross-cutting comments were liberal comments on conservative vlogger and news outlet videos or conservative comments on liberal vlogger and news outlet comments.

#### Manual analysis

The total 1,000 comments from vloggers’ channels were manually coded by two coders. The coders were trained based on a codebook in which the definitions and boundaries of the four political leanings—*conservative*, *liberal*, *other*, and *indeterminable*—were delineated; *other* was checked when there was a distinguishable political leaning in the comment, but it could not fall into either conservative or liberal; *indeterminable* was checked when the comment did not have enough information to distinguish its political leaning (see Appendix for more details about the codebook). After three training meetings, an identical set of 200 comments—100 comments from each political group—were coded by the two coders to measure their intercoder reliability score. The score was .835 in Gwet’s AC1 statistic [37], which is higher than the conventional threshold of intercoder reliability score that can be accepted, .800 [38]. With this acceptable score, the remaining 800 comments were evenly assigned to the coders. After the final coding, the proportion of cross-cutting comments in each political group (i.e., conservative comments on liberal videos or liberal comments on conservative videos) was measured. Finally, the existing labels were converted into binary classification labels—*cross-cutting* and *non-cross-cutting* comments—and a chi-square test of independence identified if the cross-cutting proportion significantly varied by the vlogger’s political leaning.

#### Computational analysis

Comments from mainstream news outlets’ videos were analyzed by multiclass classification models with three labels—*conservative*, *liberal*, and *neither of them* (hereinafter *neither*)—based on supervised learning. The vlogger comments and coding results of the manual analysis were utilized as training data. Whereas the number of labels was four (*conservative*, *liberal*, *other*, and *indeterminable*) in the original manual analysis, the two categories, *other* and *indeterminable*, were combined into *neither* in this computational analysis because the number of labels is significantly related to the accuracy of classification models. For instance, Schwarz’s study [35], which is another study that used computational methods to analyze YouTube comments about the Mueller report, initially used three labels (conservative, liberal, neither) but converted them into two labels (conservative, liberal) since binary classification models can have better accuracy scores. We also faced the same challenging situation but decided to stick to multiclass classification models. While this approach made us have relatively lower accuracy scores, we believe that there are many YouTube comments that are neither conservative nor liberal and that without a practically acceptable set of labels, our findings could be somewhat pointless.

Every process in this part of the analysis was conducted via Python. First, all comments in our sample went through a preprocessing stage; using the popular NLP library spaCy [39], all 326 stop words were removed, and tokenization was conducted. In the embedding stage, one of the most state-of-the-art embedding models, Sentence Embeddings using Siamese BERT (SBERT), which is a modified BERT network that incorporates Siamese and triplet networks to produce semantically meaningful sentence embeddings, was used [40]; specifically, the *all-mpnet-base-v2* sentence-transformer model was employed based on its outstanding performance scores. Using the embedding results, we built three supervised models, which utilized three different machine learning algorithms—logistic regression, support vector machine (SVM), and random forest—all of which are commonly used in the relevant research literature [e.g., 41–43].

Logistic regression is one of the most common machine learning algorithms for classification tasks, and it uses a Sigmoid function (logistic function) to predict the output when the outcome variable is categorical [44]. SVM is another common algorithm used for classification. Briefly speaking about its mechanism, the algorithm finds a hyperplane (i.e., a decision boundary in the vector space) that can divide data points into two groups with the highest margin [45]. Different from the previous two algorithms, the last one, random forest, is an ensemble algorithm; the algorithm builds multiple decision trees at the training stage and selects the class that the highest number of trees indicated at the classification stage. This mechanism is commonly used when analyzing high-dimensional data and observing complicated relationships [46]. It is worth noting that we initially considered employing later-developed NLP models than these three. However, due to our limited sample size and possible overfitting issues, we concluded that examining these three relatively traditional, but still popular, models would be a more appropriate choice. In fact, while these three algorithms have been used with one another in previous studies [47,48], few have employed them together in the context of political YouTube comments and compared their performances. Therefore, it can be another methodological contribution of this study to identify, among the three machine learning algorithms, which one is the most accurate for classifying the political leanings of YouTube comments.

The accuracy scores of the three models were measured by comparing their results with human-coding results. To that end, first, 200 comments were randomly drawn from the total of 4,230 comments from mainstream news outlet videos. Then, those comments were classified by both the three models and one human coder, who was one of the two coders in the previous manual analysis. Next, the accuracy, macro-F1, macro-precision, and macro-recall scores of the three models were calculated based on the human-coding results; macro averaging is one of the most simple and common ways to calculate F1, precision, and recall scores of multiclass classifiers, which is calculated using the unweighted mean of all class-level scores. After that, only the models whose accuracy scores were over the baseline (.333) could move to the next stage, which predicted all of the remaining 4,030 comments on the mainstream news outlet videos. After the classification process, the proportion of comments with each political leaning was calculated for each of the three mainstream new outlet groups—conservative, liberal, and neutral. Specifically for the neutral news outlet, the ratio between conservative and liberal comments was computed. Finally, after converting the existing labels to binary classification labels—*cross-cutting* and *non-cross-cutting*—it was tested if the cross-cutting proportion significantly varied between the conservative and liberal news outlet groups via a chi-square test.

To address RQ4, the proportions of cross-cutting comments on mainstream news outlet videos were compared to those on vlogger videos. In this analysis, only one of the three classification models that showed the highest accuracy score was used for the comparison. Again, after converting the existing labels to the binary classification labels (*cross-cutting* vs. *non-cross-cutting*), it was examined if the cross-cutting proportion significantly varied between vlogger and mainstream news outlet videos via a chi-square test.

## Results

### Vlogger comments

As Table 3 shows, of the 500 comments from conservative vlogger videos, 381 comments (76.2%) showed *conservative* leanings, while 15 comments (3.0%) showed *liberal* leanings. There were 20 comments (4.0%) for *other* and 84 comments for *indeterminable* (16.8%). When it comes to liberal vlogger videos, 350 comments (70.0%) showed *liberal* leanings, while 50 comments (10.0%) showed *conservative* leanings. There were 5 comments (1.0%) for *other* and 84 comments (19.0%) for *indeterminable*. Taken together, the proportion of cross-cutting comments in the liberal vlogger video group (10.0%) is about 3.3 times higher than that in the conservative vlogger video group (3.3%). A chi-square test showed that the difference was statistically significant, *X*^2^ (1, *N* = 1000) = 19.02, *p* < .001.

**Table 3:** Political leaning distribution of comments on vlogger videos.

	Political leaning of comment				
	Conservative	Liberal	Other	Indeterminable	Total
**Political leaning of vlogger**					
Conservative	381 (76%)	15 (3%)	20 (4%)	84 (17%)	500 (100%)
Liberal	50 (10%)	350 (70%)	5 (1%)	95 (19%)	500 (100%)

### Mainstream news outlet comments

Of the three models—logistic regression, SVM, and random forest—all of which were trained with the manual analysis results, the logistic regression model showed the highest accuracy score (.640) regarding its prediction of the 200 random comments from mainstream news outlet videos, followed by the SVM model (.615) and the random forest model (.610). When it comes to the macro-F1 score, again, the highest one was the logistic regression model (.578), followed by the SVM (.558) and the random forest (.439). Table 4 also displays the precision and recall scores for each model. For any metric, the logistic regression showed the best performance, and the SVM and random forest followed it. All these three models’ accuracy scores were considerably higher than the baseline (.333); the logistic regression, SVM, and random forest models performed 92%, 85%, and 83% better than the baseline. Hence, all three models moved to the next stage, which predicted the political leanings of the 4,030 remaining comments on the mainstream news outlet videos.

**Table 4:** Performance scores of the three models.

	Logistic regression	SVM	Random forest
Accuracy	.640	.615	.610
Macro-F1	.578	.558	.439
Macro-precision	.623	.567	.562
Macro-recall	.568	.567	.446

#### Logistic regression

Table 5 exhibits the final results of the total 4,230 comments predicted by the logistic regression model. The logistic regression model predicted that of the 2,421 comments from conservative news outlets, 53.0% (1,282 comments) were *conservative*, while *liberal* and *neither* comprised 39.2% (949 comments) and 7.9% (190 comments), respectively. Of the 1,672 comments from liberal news outlets, the model predicted that 54.4% (909 comments) were *conservative*, followed by *liberal* (42.1%, 704 comments) and *neither* (3.5%, 59 comments). Lastly, of the 137 comments from the neutral news outlet, the model predicted that 46.7% (64 comments) were *conservative*, followed by *liberal* (45.3%, 62 comments) and *neither* (8.0%, 11 comments); the ratio between the conservative and liberal comments was 1.03:1.00. A chi-square test showed that the political leaning of comments significantly varied by the news outlet’s political leaning, *X*^2^ (4, *N* = 4230) = 35.20, *p* < .001. When it came to cross-cutting comments, the liberal video group’s cross-cutting proportion (54.4%) was 1.4 times higher than the conservative group’s (39.2%), and the difference was statistically significant, *X*^2^ (1, *N* = 4093) = 91.17, *p* < .001.

**Table 5:** Prediction results by the logistic regression model.

	Political leaning of comment			
	Conservative	Liberal	Neither	Total
**Political leaning of news outlet**				
Conservative	1282 (53%)	949 (39%)	190 (8%)	2421 (100%)
Liberal	909 (54%)	704 (42%)	59 (4%)	1672 (100%)
Neutral	64 (47%)	62 (45%)	11 (8%)	137 (100%)

#### SVM

Table 6 displays the final results of the total 4,230 comments predicted by the SVM model. Of the 2,421 comments from conservative news outlets, the SVM regression model predicted that 48.5% (1,175 comments) were *conservative*, followed by *liberal* (39.0%, 943 comments) and *neither* (12.5%, 303 comments). Of the 1,672 comments from liberal news outlets, the model predicted that 49.5% (827 comments) were *conservative*, followed by *liberal* (43.7%, 730 comments) and *neither* (6.9%, 115 comments). Lastly, of the 137 comments from the neutral news outlet, the model predicted that both *conservative* and *liberal* comments identically comprised 45.3% (62 comments), and *neither* comprised 9.5% (13 comments); thus, the ratio between the conservative and liberal comments was 1.00:1.00. A chi-square test showed that the political leaning of comments significantly varied by the news outlet’s political leaning, *X*^2^ (4, *N* = 4230) = 37.42, *p* < .001. When it came to cross-cutting comments, the liberal video group’s cross-cutting proportion (49.5%) was 1.3 times higher than the conservative group’s (39.0%), and the difference was statistically significant, *X*^2^ (1, *N* = 4093) = 44.09, *p* < .001.

**Table 6:** Prediction results by the SVM model.

	Political leaning of comment			
	Conservative	Liberal	Neither	Total
**Political leaning of news outlet**				
Conservative	1175 (49%)	943 (39%)	303 (13%)	2421 (100%)
Liberal	827 (50%)	730 (44%)	115 (7%)	1672 (100%)
Neutral	62 (45%)	62 (45%)	13 (9%)	137 (100%)

#### Random forest

Table 7 displays the final results of the total 4,230 comments predicted by the random forest model. Of the 2,421 comments from conservative news outlets, the random forest model predicted that 71.5% (1,730 comments) were *conservative*, while the *liberal* and *neither* comprised 27.4% (663 comments) and 1.2% (28 comments), respectively. Of the 1,672 comments from liberal news outlets, the model predicted that 66.6% (1113 comments) were *conservative*, followed by *liberal* (32.6%, 545 comments) and *neither* (0.8%, 14 comments). Lastly, of the 137 comments from the neutral news outlet, the model predicted that 62.0% (85 comments) were *conservative*, followed by *liberal* (37.2%, 51 comments) and *neither* (0.7%, 1 comment); the ratio between the conservative and liberal comments was 1.67:1.00. A chi-square test showed that the political leaning of comments significantly varied by the news outlet’s political leaning, *X*^2^ = 17.25, *p* = .004; we computed the *p*-value by Monte Carlo simulation (based on 2,000 replicates) in this chi-square calculation since one of the cells had a very low value. When it came to cross-cutting comments, the liberal video group’s cross-cutting proportion (66.6%) was 2.4 times higher than the conservative group’s (27.4%), and the difference was statistically significant, *X*^2^ (1, *N* = 4093) = 616.52, *p* < .001.

**Table 7:** Prediction results by the random forest model.

	Political leaning of comment			
	Conservative	Liberal	Neither	Total
**Political leaning of news outlet**				
Conservative	1730 (72%)	663 (27%)	28 (1%)	2421 (100%)
Liberal	1113 (67%)	545 (33%)	14 (1%)	1672 (100%)
Neutral	85 (62%)	51 (37%)	1 (1%)	137 (100%)

### Vlogger comments vs. mainstream news outlet comments

For this part of the analysis, we used results from the mainstream news outlet comments predicted by only the model with the highest accuracy score, which was the logistic regression model. Comparing the cross-cutting proportions between the *conservative* vlogger and mainstream news outlet groups, the proportion in the conservative mainstream news outlet group was 39.2%, and the value was about 13 times higher than the proportion in the conservative vlogger group (3.0%). The difference was statistically significant, *X*^2^ (1, *N* = 2921) = 91.17, *p* < .001. When it came to the cross-cutting proportion comparison between the *liberal* vlogger and mainstream news outlet groups, the proportion in the liberal mainstream news outlet group was 54.4%, and the value was about 5 times higher than the proportion in the liberal vlogger group (10.0%). This difference was also statistically significant, *X*^2^ (1, *N* = 2172) = 243.96, *p* < .001. Taken together, regardless of the channel’s political leaning, mainstream news outlet comment threads had a significantly higher proportion of cross-cutting comments than vlogger comment threads.

## Discussion

### Cross-cutting discussions on vlogger videos

Our manual analysis of comments on vlogger videos revealed that cross-cutting discussions are more common in liberal vloggers’ comment threads than in conservative vloggers’. These results confirmed Heatherly et al.’s [13] findings about the difference in SNS cross-cutting discussions between conservatives and liberals. In addition, our results are in line with another YouTube study by Wu and Resnick, which found that “conservatives were much more likely to comment on left-leaning videos than liberals on right-leaning videos” [14, p. 808]. Given that they did not categorize the vlogger group as an independent group, the current study newly suggests that the asymmetric cross-cutting discussion pattern between general conservative and liberal channels is valid particularly on political vlogger channels. Moreover, our findings are also connected to those of Chae and Hara’s recent study [31]. They found that after watching a conservative news outlet video, a liberal news outlet video—which may be opinion-challenging information—is more likely to be recommended by the YouTube algorithm; meanwhile, after watching a liberal news outlet video, another liberal news outlet video—which may be opinion-reinforcing information—is more likely to be recommended. Granted, the authors clarified that the YouTube recommendation algorithm can be both a cause and effect of users’ behavior, but our results show that future research should further explore the recommendation network of political vlogger videos to understand how the network is associated with cross-cutting discussions on those videos.

Our comparison between comments on vlogger and mainstream news outlet videos revealed that cross-cutting comments were less often shown in political vlogger comment threads than in mainstream news outlet threads. This finding corresponds to Wu and Resnick’s suggestion that mainstream media news outlet videos have higher rates of cross-cutting discussions than videos uploaded by other types of news media on YouTube [14]. Although we cannot identify why vlogger comments have relatively fewer cross-cutting discussions within the design of the current study, here, we share our best guess based on prior literature. Recent literature on communication on YouTube shows that there are parasocial relationships between YouTube personalities and their viewers, which means that loyal viewers consider YouTubers like their real-world friends [49]. Our findings could suggest that the parasocial relationships between general YouTube influencers and their viewers are also valid specifically between political vloggers and their viewers. In other words, while viewers of mainstream news outlet channels perceive the uploader as a media channel, viewers of political vlogger channels might perceive the uploader as more of a friend. Accordingly, political vloggers’ strong intimacy with their viewers could induce their viewers to move their political stance in a more identical way with the vloggers’. Granted, it is likely that the viewers’ political leanings might correspond to the political vlogger’s from the beginning, as the notion of selective disclosure suggests that people are more likely to be drawn to opinion-reinforcing ideas [9]. However, it is possible that their political leanings are reinforced by emotional empathy with their persona (i.e., the political vlogger) in the parasocial relationships [50] or by a course of social learning via watching the vlogger’s videos regularly [51].

The parasocial relationships between political vloggers and their viewers can make more serious influence on our society compared to other types of parasocial relationships on YouTube. Our findings showed that echo chambers can be more easily fostered with political vlogger videos than with mainstream news outlet videos. While viewers’ tendency to have similar political ideas with vloggers’ is not problematic in and of itself, the problem is that some popular political vloggers on YouTube share extreme political opinions [52]. Accordingly, their extreme political leanings can lead their viewers to also have extreme political notions and confine themselves into an echo chamber. This risk is also connected to concerns about online fandoms or communities with extreme community culture, which have been discussed by previous social media studies. For instance, Seering et al. [53] cautioned about the autonomy of online communities, which can encourage a distorted worldview, such as misogyny or racism, depending on the community culture.

We believe that our finding of a lack of cross-cutting discussions on political vlogger videos should serve as a wake-up call. There is a risk that political vlogger videos can become a new venue to facilitate political polarization. Especially in the U.S., where political partisanship has become a serious social issue, communication on political vlogger videos requires more society-level attention. Accordingly, future research needs to extend this work and investigate if political vloggers’ channels or communities function as the epicenter of political polarization through network analysis or other research methods.

#### Methodological utility of political vlogger comments

The current study also methodologically tested the utility of political vlogger comments as a source of training data in NLP based on supervised learning. We employed political vlogger comments as training data to train multiclass classification models for predicting the political leanings of comments on mainstream news outlet videos. Since we decided to develop multiclass classification models with three labels (conservative, liberal, and neutral) rather than adjusting them to binary models, the accuracy scores of our models were expected to be low. Considering these conditions, the actual accuracy scores of the three models were fairly high (logistic regression: .640, SVM: .615, random forest: .610); the scores of all three models were more than 1.8 times higher than the baseline (.333). In fact, the accuracy scores of our three models are higher than those of the two binary classification models in Schwarz’s study [35], which also predicted the political leanings of YouTube comments about the Mueller report using NLP. Interestingly, those two models are the only two that did not use YouTube comments as training data but other sources—presidential candidates’ tweets and Manifesto Corpus of political speech—in the study. This may denote that when predicting the political leanings of social media text via supervised learning, it is important to collect training data from the same social media platform to gain a better accuracy score. While the accuracy scores of our three models are still far from perfect, we believe that this study opens the possibility that political vlogger videos can be utilized as a good source to predict political leanings of comments on other types of videos.

### Cross-cutting discussions on mainstream news outlet videos

Our computational analysis of comments on mainstream news outlet videos revealed that there are a large quantity of cross-cutting discussions in both conservative and liberal mainstream news outlet comment threads. Our findings about comments on mainstream news outlet videos also supported the asymmetry that liberal videos have significantly more cross-cutting comments than conservative videos, which corresponds not only to prior literature [13,14] but also to the results of the vlogger comment analysis in the current study. Again, the proportion of cross-cutting comments in the mainstream news outlet comment threads was higher than that in the vlogger comment threads, regardless of the channel’s political leaning. Via our additional qualitative observations of mainstream news outlet videos, we found some clues to help explain this difference. Some of the cross-cutting comments explicitly ridiculed mainstream news outlets and their opposite political groups (e.g., “You guys HAVE NOTHING……GAME F*CKIN OVER B*TCHES” in the CNN thread). Put differently, for users who want to ridicule the opposite group, mainstream news outlet videos are good targets for attack, and their commenting behavior sometimes even becomes troll-like (e.g., “CNN on suicide watch” in the CNN thread).

This observation leads us to a more fundamental question: Do cross-cutting comments really enhance our political communication? Previous studies on cross-cutting discussions consider it to be a possible solution to the echo chamber risk [13]. However, as the above example demonstrated, a cross-cutting discussion, in and of itself, is not necessarily beneficial to productive political communication. Therefore, the current study suggests that the proportion of cross-cutting comments should not necessarily be interpreted as an index to represent the diversity of political opinions. Furthermore, our qualitative observations provide an important caveat that cross-cutting discussions should not be observed solely by a quantitative method, including NLP methods, but additional qualitative observations are vital to avoid any misleading findings.

In fact, both the quantitative and qualitative observations indicate that the most promising news outlet group where conservatives and liberals can gather and share their ideas in a productive manner was neither the conservative nor liberal news outlet channels, but the neutral news outlet channel, C-SPAN. In terms of the quantified proportions, all three models suggested that the C-SPAN comment thread contained the most proportionate number of conservative and liberal comments. The SVM model even predicted that the proportions of conservative and liberal comments in the C-SPAN comment thread were exactly the same (45.3%). Thus, it is highly possible that a similar number of conservatives and liberals mixed together in the politically neutral news outlet video. More importantly, the quality of the comments in the C-SPAN thread showed that conservatives and liberals did not come to the video to ridicule their opposite political group since the news outlet does not have a clear political leaning, but rather the contents of their comments were relatively rational (e.g., “Guys all this name-calling in character bashing is not helpful. We need to focus on what’s really important right now.”). Accordingly, the current study suggests the possibility of neutral news media outlets as a venue where productive discussions beyond partisanship can happen with low antipathy to the opposite political group. This possibility is also aligned with a recent finding by Chae and Hara that YouTube channels with less clear political leanings can work as a bridge between conservative and liberal videos in the YouTube recommendation network [31]. Taken together, all these findings call for more research on cross-partisan discussions on politically neutral news outlets’ social media channels/accounts.

Lastly, while it is not specifically related to our original research questions, it is worth mentioning that the current study found that the *context of the news* could be significantly associated with the political leanings of comments on mainstream news. Across the results of the three classification models, it was found in common that conservative comments were more abundant than liberal comments regardless of the news outlet’s political leaning, which means that even in the liberal news outlets’ comment threads, there were more conservative comments than liberal comments. This finding was somewhat surprising; we would expect more liberal comments than conservative comments in a liberal channel’s comment thread. That said, when considering the context of the political situation at the time when the news about Mueller’s report submission was released, which was clearly favorable to conservatives, the overwhelming proportion of conservative comments is understandable. Most conservative commenters posted their comments on the conservative news outlet videos to celebrate their victory (e.g., “Congratulations. One step closer to absolute power 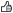
” and “Great day for all law-abiding and patriotic Americans” in the Fox News thread) and commented on the liberal news outlet videos to ridicule the liberal news outlets and liberal viewers (e.g., “Haha CNN eat Trump’s sh*t” and “CNN FAKE NEWS!!!” in the CNN thread). Meanwhile, liberals might have been reluctant to talk about the news as it was not positive for them nor something they wanted to discuss further in the comment thread. Altogether, while it seems implausible that liberal news outlet videos have more conservative comments than liberal comments in general cases, this study showed that depending on the news context, it is possible.

Interestingly, the same did not go for vlogger comments. Despite the news context at the time, the dominant political leaning in the vlogger comment threads always coincided with the political leaning of the vlogger, which is starkly contrasting with the results of the mainstream news outlet video analysis. Put differently, political leanings in vlogger comment threads seem less susceptible to the news context. This difference between vlogger and mainstream news outlet videos is understandable, considering that political vlogger channels might not have a sudden influx of viewers because of a certain news context since their channels are not very well-known to represent particular political groups like mainstream news outlets (e.g., Fox News, MSNBC), but rather stably keep their viewer group as more of a community. This also corresponds to prior literature suggesting YouTube personalities’ fandom communities [54]. We hope that future research can shed more light on this interesting topic with a specific research design to further investigate this difference between vlogger and mainstream news outlet channels.

### Limitations

As with any study, this study has some limitations that should be noted. Above all, our findings are limited in terms of their generalizability. The basic assumption of this study is that the utility of NLP for political leaning analysis is valid only when the texts were written in a homogeneous context. On account of this reason, we did not expand our topic range beyond one particular topic, the Mueller report. However, an avoidable limitation that accompanies this approach is that our findings may not be applicable to other cases where the context is significantly different. Another notable limitation of this study is that we conducted our analysis of comments on mainstream news outlet videos using the NLP models whose accuracies were limited. Especially since our models are multiclass classification models, their accuracy scores could not reach an ideal level, while all their scores were considerably higher than the baseline. Therefore, although our results about mainstream news outlet comments were overall consistent across the three different models, they should be interpreted with caution given the limited accuracies of the models. Lastly, due to its limited research design, we did not take into account the community culture of each vlogger channel in this study. Given prior literature highlighting the importance of community culture on YouTube [55], we believe that this is where future research could go further by particularly focusing on this topic.

## Conclusion

The current study examines comments from political vlogger videos and mainstream news outlet videos on YouTube using both manual and computational analyses to identify how cross-cutting discussions vary by a channel’s political leaning and media type. As a result, we found that the proportion of cross-cutting comments significantly varies by the channel’s political leaning. More specifically, our results confirm the findings of prior literature that there are more cross-cutting discussions on liberal YouTube channels than on conservative YouTube channels [14]. In addition, this study shows the possibility of neutral news media outlets as a venue for constructive cross-cutting discussion beyond partisanship. When it comes to media type, we found that mainstream news outlet videos have significantly more cross-cutting discussions than political vlogger videos regardless of the channel’s political leaning. Additionally, this study also found the possibility that comments on vlogger videos can be utilized as a source of training data to classify political leanings of comments from other types of channels. Lastly, our qualitative observations provided two meaningful points. First, a considerable proportion of cross-cutting comments posted to simply ridicule the opposite political group made us question if cross-cutting comments were conducive to productive political communication. Second, when analyzing the political leanings of comments on online/social media news, it is important to consider the news context to better interpret the results.

Although this study has some limitations, it expands our understanding of cross-cutting discussions on video-based social media. We hope that our work can be a useful stepping stone to a variety of future research on this topic.

## Supporting information

S1 File Appendix: Coding instrument for political view. (PDF)
