# Supplementary material for: Where do cross-cutting discussions happen?: Identifying cross-cutting comments on YouTube videos of political vloggers and mainstream news outlets
Source: PLoS One. 2024 May 29;19(5):e0302030. doi: 10.1371/journal.pone.0302030 (PMC11135730; doi:10.1371/journal.pone.0302030)
Supplement: S1 File — (PDF) [file pone.0302030.s001.pdf]

Appendix: Coding instrument for political view

You are being asked to distinguish the political views of YouTube comments and record those results in the column "political view" in the given Excel sheet. There are four categories you can select in this variable: "conservative," "liberal," "other," and "indeterminable." Each of these categories should be mutually exclusive and coded based on the following definitions/criteria.

Conservative

In the current coding, *conservative* is defined as a political view developing "relatively favorable opinions about existing institutions and authorities and to dismiss or reject the possibility of change, especially in its more radical forms" [1, p. 272]. In the context of U.S. politics, this political view generally corresponds to Republicans'; thus, when the message of a comment in this coding is in line with Republicans' general ideas, this category is present. However, this category is also present when the message is not aligned with Republicans' general ideas but corresponds to the above definition of a conservative political view.

Liberal

In the current coding, *liberal* is defined as a political view exhibiting "stronger preferences for social change and equality (as well as progress and flexibility over tradition and stability, respectively)" [2, p. 312]. In the context of U.S. politics, this political view generally corresponds to Democrats'; thus, when the message of a comment in this coding is in line with Democrats' general ideas, this category is present. However, this category is also present when the message is not aligned with Democrats' general ideas but corresponds to the above definition of a liberal political view.

Other

In the current coding process, *other* is present when a comment discloses the commenter's political view, but the view is not in line with either *conservative* or *liberal*.

Indeterminable

In the current coding process, *indeterminable* is present in every case where there is not enough information to distinguish the political view of the comment.

References

1. Jost JT, Fitzsimons G, Kay AC. The ideological animal. In: Greenberg J, Koole SL, Pyszczynski TA, editors. Handbook of Experimental Existential Psychology. New York: Guilford Press; 2004. p. 263-83.
2. Jost JT, Federico CM, Napier JL. Political ideology: Its structure, functions, and elective affinities. Annual Review of Psychology. 2009; 60:307-37.
